# Defining the Dynamic Regulation of O-GlcNAc Proteome in the Mouse Cortex---the O-GlcNAcylation of Synaptic and Trafficking Proteins Related to Neurodegenerative Diseases

**DOI:** 10.3389/fragi.2021.757801

**Published:** 2021-09-29

**Authors:** Van N Huynh, Sheng Wang, Xiaosen Ouyang, Willayat Y Wani, Michelle S Johnson, Balu K Chacko, Anil G Jegga, Wei-Jun Qian, John C Chatham, Victor M Darley-Usmar, Jianhua Zhang

**Affiliations:** ^1^ Department of Pathology, Center for Free Radical Biology, University of Alabama at Birmingham, Birmingham, AL, United States; ^2^ Biological Sciences Division, Pacific Northwest National Laboratory, Richland, WA, United States; ^3^ Division of Biomedical Informatics, Cincinnati Children’s Hospital Medical Center, Department of Pediatrics, University of Cincinnati College of Medicine, Cincinnati, OH, United States; ^4^ Department Veterans Affairs, University of Alabama at Birmingham, Birmingham, AL, United States

**Keywords:** OGA, O-GlcNAc, thiamet G, mass spectrometry, PICALM, DnaJC6

## Abstract

O-linked conjugation of ß-N-acetyl-glucosamine (O-GlcNAc) to serine and threonine residues is a post-translational modification process that senses nutrient availability and cellular stress and regulates diverse biological processes that are involved in neurodegenerative diseases and provide potential targets for therapeutics development. However, very little is known of the networks involved in the brain that are responsive to changes in the O-GlcNAc proteome. Pharmacological increase of protein O-GlcNAcylation by Thiamet G (TG) has been shown to decrease tau phosphorylation and neurotoxicity, and proposed as a therapy in Alzheimer’s disease (AD). However, acute TG exposure impairs learning and memory, and protein O-GlcNAcylation is increased in the aging rat brain and in Parkinson’s disease (PD) brains. To define the cortical O-GlcNAc proteome that responds to TG, we injected young adult mice with either saline or TG and performed mass spectrometry analysis for detection of O-GlcNAcylated peptides. This approach identified 506 unique peptides corresponding to 278 proteins that are O-GlcNAcylated. Of the 506 unique peptides, 85 peptides are elevated by > 1.5 fold in O-GlcNAcylation levels in response to TG. Using pathway analyses, we found TG-dependent enrichment of O-GlcNAcylated synaptic proteins, trafficking, Notch/Wnt signaling, HDAC signaling, and circadian clock proteins. Significant changes in the O-GlcNAcylation of DNAJC6/AUXI, and PICALM, proteins that are risk factors for PD and/or AD respectively, were detected. We compared our study with two key prior O-GlcNAc proteome studies using mouse cerebral tissue and human AD brains. Among those identified to be increased by TG, 15 are also identified to be increased in human AD brains compared to control, including those involved in cytoskeleton, autophagy, chromatin organization and mitochondrial dysfunction. These studies provide insights regarding neurodegenerative diseases therapeutic targets.

## Introduction

First discovered in the 1980s, the O-GlcNAcylation of proteins is now widely accepted as playing a key role in the regulation of diverse biological processes integrating nutrient availability and cellular stress (Zachara and Hart, 2004; Love and Hanover, 2005; Slawson et al., 2006; Paruchuri and Zachara, 2011; Darley-Usmar et al., 2012; Bond and Hanover, 2013; Alonso et al., 2014; Hardivillé and Hart, 2014; Marsh et al., 2014; Wani et al., 2015). System biology studies implicate perturbation of O-GlcNAc transferase (OGT) as one of the nine genes identified to impact transcriptome regulation in similar fashion as caloric restriction/intermittent fasting (Hou et al., 2016). Changes in O-GlcNAc levels have been linked to aging and a number of neurodegenerative diseases (Zhang, 2020; Mueller et al., 2021). For example, several investigators reported age-dependent changes of overall protein O-GlcNAcylation in animal models (Fülöp et al., 2008; Liu et al., 2012). However, there is substantial controversy as to the outcome and mechanisms involved. In human postmortem brains, total O-GlcNAcylation has been found to increase in Alzheimer’s disease (AD) brains in detergent-insoluble fractions (Griffith and Schmitz, 1995). A recent more comprehensive proteomics study has reported dynamic changes in protein O-GlcNAcylation in AD patients with 12 peptides decreased and 119 peptides increased compared to controls (Wang et al., 2017).

In animal models, pharmacological approaches to increase O-GlcNAc levels, such as inhibition of O-GlcNAcase (OGA) by Thiamet G (TG), increased tau O-GlcNAcylation and decreased tau phosphorylation and associated neurodegenerative phenotypes (Yuzwa et al., 2008; Yuzwa et al., 2012; Yuzwa et al., 2014; Zhu et al., 2014; Hastings et al., 2017). These putative beneficial effects of increasing tau O-GlcNAc modification suggest a potential utility of OGA inhibitors such as TG or its derivatives, for treatment of AD. However, we have shown that in aged rats overall brain O-GlcNAc levels were increased compared to younger animals (Fülöp et al., 2008), suggesting a dysregulation in O-GlcNAcylation occurs with aging, which is a well-established risk factor for AD. Moreover, we have demonstrated that increases in O-GlcNAc levels *in vivo* impaired learning and memory (Taylor et al., 2014).

In the context of PD, the impact of O-GlcNAcylation is also complex. For example, recent studies have reported that increased O-GlcNAc modification of α-synuclein blocks its aggregation and toxicity *in vitro* (Marotta et al., 2012; Marotta et al., 2015; Lewis et al., 2017). However, in a *C. elegans* model of neurodegeneration, the OGA inactive mutant, which is associated with increased O-GlcNAc levels, has been shown to increase proteotoxicity (Wang et al., 2012). Interestingly, we found a significant increase in overall protein O-GlcNAcylation levels in postmortem brain specimens of PD patients compared to control (Wani et al., 2017). Our recent studies have shown that in primary neurons, pharmacologically increased neuronal O-GlcNAc levels by TG enhanced MTOR phosphorylation, suppressed autophagy and increased α-synuclein accumulation (Wani et al., 2017). Considering these interesting and complex observations, a better understanding of the O-GlcNAc proteome and the effects of OGA inhibition are needed.

There are several approaches that have been used to catalog the brain O-GlcNAcylated proteins and O-GlcNAc proteomes. For example, wheat germ agglutinin (WGA)-based lectin weak affinity chromatography (LWAC) was used to enrich O-GlcNAc-modified peptides that were then identified by mass spectrometry. The first of these studies revealed 145 unique O-GlcNAcylated peptides in mouse brain postsynaptic preparations (Vosseller et al., 2006). Using the same enrichment approach with mouse synaptic membrane preparations and high pH reverse phase chromatography, one study identified over 1750 O-GlcNAcylation sites in 676 proteins (Trinidad et al., 2012). The O-GlcNAcylation process does not appear to be indiscriminate or always reciprocal to phosphorylation. For example, 46 Ser or Thr phosphorylation sites were identified on the microtubule associated protein tau which is extensively phosphorylated in animal models of AD, yet no tau O-GlcNAcylation was found (Trinidad et al., 2012). In addition, it appears that OGT and kinases do not favor the same amino acid residues and statistically the O-GlcNAcylation and phosphorylation sites are independent of one another. Indeed, there are at least 11-fold more phosphorylation than O-GlcNAcylation sites in synaptosomes (Trinidad et al., 2012). However, in cerebrocortical tissue using a different method (CEPC, see the next paragraph), it was found that O-GlcNAcylation sites were either at or near known phosphorylation sites, suggesting mutual exclusion (at the same site) or synergy (at the same site or those in close proximity) (Alfaro et al., 2012). Another study identified 463 O-glycopeptides corresponding to 122 proteins, though the modifications included both O-GlcNAcylation and other types of glycosylation (Trinidad et al., 2013). A similar approach was utilized in a more recent study, which identified 926 and 919 O-GlcNAcylated proteins in substantia nigra - ventral tegmental area, and the striatum, respectively (Lee et al., 2020). Although without any information regarding the sites of modification, 26 proteins were increased and 11 proteins with decreased O-GlcNAcylation levels in the dopaminergic neuron-specific *Oga* knockout mice compared to control (Lee et al., 2020).

Using a chemoenzymatic approach with an unnatural UDP substrate and Y289L GalT enzyme, coupled with biotinylation and avidin enrichment, an earlier study identified 25 O-GlcNAcylated proteins from the rat forebrain (Khidekel et al., 2004). Later studies used similar chemoenzymatic approaches coupled with photochemical cleavage (CEPC) to enrich O-GlcNAc peptides, which enhances analytical sensitivity by tagging the peptides with a photochemical cleavable-biotin probe and have a low false rate due to the specific enzymatic chemical reactions. One such study discovered 274 O-GlcNAcylated proteins from cerebrocortical tissues of 1 year old normal and 3xTg AD mice (Alfaro et al., 2012). Several synaptic, cytoskeletal, transcriptional regulators, membrane proteins, and notably extracellular domains of membrane proteins, were identified (Alfaro et al., 2012). A comprehensive meta-analysis of 378 human studies including those focusing on overall protein O-GlcNAcylation with an O-GlcNAc antibody based approach, those using focused approaches on individual proteins, and those with mass spectrometry-based omics approach suggested that the overall human O-GlcNAcome may include more than 5000 proteins and 7,000 sites (Wulff-Fuentes et al., 2021). Due to the nature of this meta-analysis, published articles with false positives were also included (Wang et al., 2014; Ma et al., 2021a). The critically curated O-GlcNAcAtalas (https://oglcnac.org) reports 4554 proteins with unambiguous O-GlcNAc sites in different species.

In this study, we used quantitative isobaric tandem mass tag (TMT) labelling combined with CEPC method to enrich O-GlcNAcylated peptides in the mouse brain coupled to tandem mass spectrometry (LC-MS/MS) to identify proteins that are modified by O-GlcNAcylation in the mouse cortex, and to compare O-GlcNAcylation levels between mice injected with saline and with the OGA inhibitor Thiamet G (TG). We identified 506 unique O-GlcNAc peptides corresponding to 278 O-GlcNAcylated proteins in the mouse cortex from both groups. Of these, 67 were not previously identified in the two most extensive studies of the O-GlcNAcome in the mouse cerebrocortical tissues (Alfaro et al., 2012) and in the human mid frontal gyrus region (Wang et al., 2017). Furthermore, O-GlcNAc levels of 155 out of the 506 unique peptides were significantly changed (*p* < 0.05) by systemic administration of TG. O-GlcNAc modification of 85 peptides corresponding to 65 proteins increased at >1.5-fold in the TG treatment group compared to controls. Network analysis of these changes revealed that pathways involved in synaptic function are enriched.

Of note, we found that O-GlcNAcylation of DNAJC6/AUXI was increased 12-fold in response to OGA inhibition. DNAJC6 has been shown to be important for clathrin uncoating (Edvardson et al., 2012), and recent studies identified mutations in DNAJC6, which appear to be responsible for juvenile and early onset PD (Edvardson et al., 2012; Köroğlu et al., 2013; Elsayed et al., 2016; Olgiati et al., 2016). Of relevance to AD, phosphatidylinositol binding clathrin assembly protein (PICALM) (which is a highly validated risk factor in late-onset AD (Harold et al., 2009)) is O-GlcNAcylated and its O-GlcNAcylation was increased by 2-fold in response to OGA inhibition. These findings suggest that DNAJC6 and PICALM O-GlcNAcylation are potential contributing factors to neurodegenerative diseases.

## Results and Discussion

### Quantitative Profiling of O-GlcNAcylated Proteins and Peptides in Mouse Cortex

**FIGURE 1:**
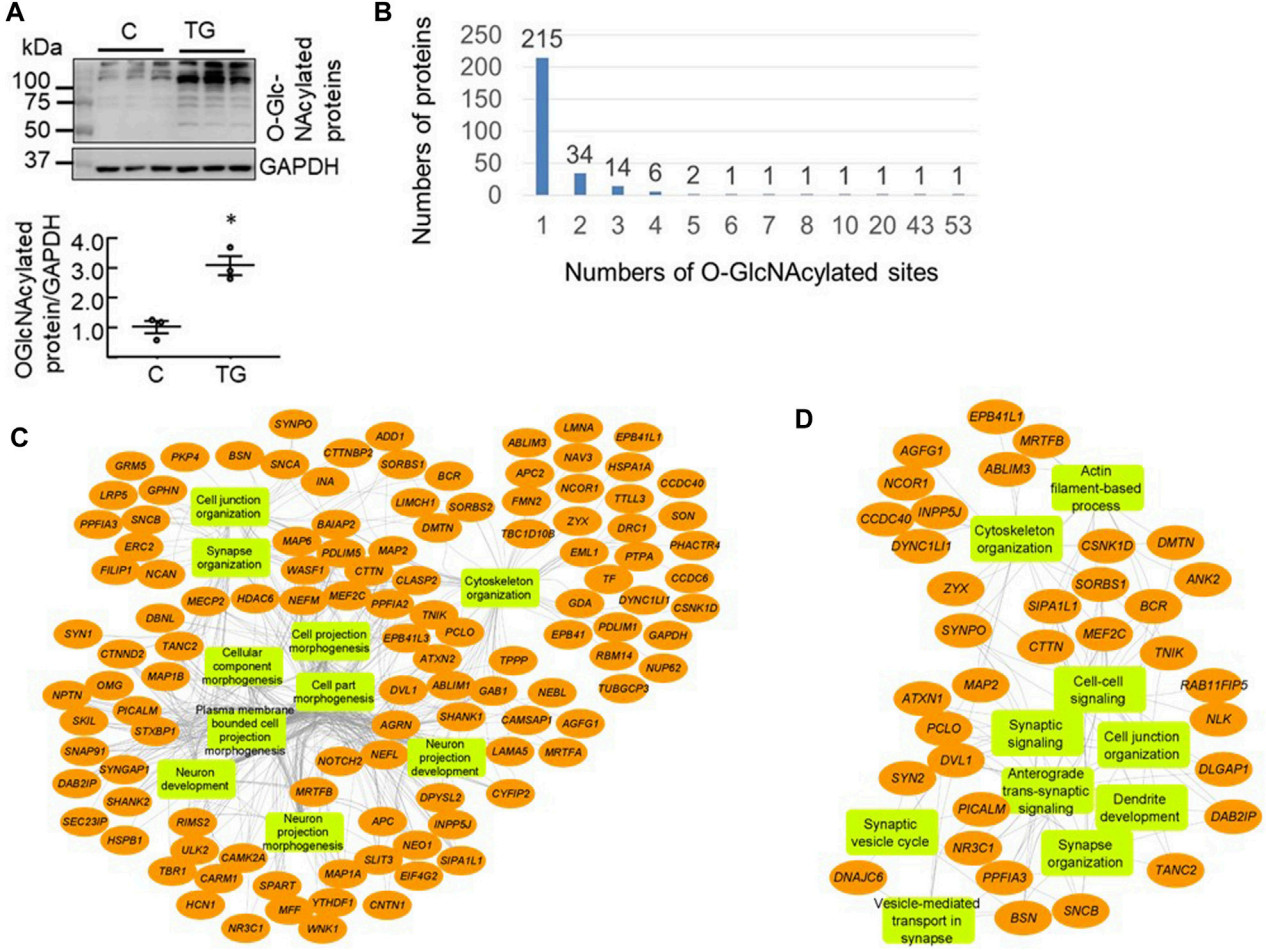
Proteomics analysis of mouse cortical O-GlcNAcome **(A)** Western blot analyses with cortical protein extracts demonstrated that 3 h post i. p. Thiamet G (TG) injection at 10 mg/kg, there was an increase of overall levels of protein O-GlcNAcylation (quantified using the entire lane) as detected by the CTD110.6 antibody (*n* = 3, *p < 0.05 Student t-test) **(B)** Using isobaric tandem mass tag labelling combined with CEPC method we identified 506 peptides corresponding to 278 proteins that were O-GlcNAcylated in the mouse cortex (*n* = 3 for control and *n* = 2 for TG). Of these, 215 proteins have one modification site, 34 proteins have two sites, 14 proteins have 3 sites, six proteins have four sites, two proteins have five sites, and one protein each has 7, 8, 10, 20, 43, and 53 sites **(C**, **D)** Network visualization of the top 10 Gene Ontology biological process terms enriched for the total 278 O-GlcNAcylated proteins identified in both Saline and TG groups (C), and the 65 proteins that exhibited significant increases in O-GlcNAcylation levels following acute TG treatment **(D)**.

To detect the proteins undergoing dynamic changes in O-GlcNAcylation we injected mice (i.p.) with saline or the OGA inhibitor TG (10 mg/kg) which inhibits O-GlcNAc removal and elevates overall levels of protein O-GlcNAcylation. Three hours after injection, we harvested half of the cortex for western blot (n = 3 each control versus TG) and half for proteomics analyses to assess the global impact on protein O-GlcNAcylation. Using hemi-cortical extracts, we found that there was a significant increase of the overall protein O-GlcNAcylation as detected by the CTD110.6 antibody (Figure 1A). There were limited numbers of bands (<10 bands) that were visible and ∼5 bands exhibited higher intensity in the TG group compared to the saline group. The patterns obtained by using western blot methodology for O-GlcNAc proteins are strongly representative of the high abundance of some O-GlcNAcylated proteins and, as is evident from the mass spectrometry analysis, a small subset of the total O-GlcNAc proteome.

Using the chemoenzymatic photocleavage (CEPC) approach coupled with TMT labeling and LC-MS/MS analyses (Alfaro et al., 2012; Wang et al., 2014; Wang et al., 2017), we identified 506 unique O-GlcNAcylated peptides corresponding to 278 proteins in cortex samples from mice administered either saline or TG (Supplementary Table S1). Figure 1B shows the distribution of the number of O-GlcNAcylated sites among the detected proteins. Some proteins have multiple O-GlcNAcylated sites whereas the majority have only one or two detected sites of modification. Notably, the proteins Bassoon (BSN) and Piccolo (PCLO) have 53 and 43 O-GlcNAcylated sites, respectively (Figure 1B).

To assess which biological pathways are affected by protein O-GlcNAcylation, we used ToppGene tools (Shannon et al., 2003) for pathway enrichment analysis and Cytoscape (Asanuma et al., 2006; Guo et al., 2018; Ji et al., 2019) to visualize the genetic network. We found that the identified 278 O-GlcNAcylated proteins are highly enriched for cellular component morphogenesis, cytoskeleton organization, synapse organization, and cell projection morphogenesis (Figure 1C). Furthermore, the identified O-GlcNAcylated proteins are associated with key neuronal cellular components including synapse and dendrite.

### Synaptic and Trafficking Proteins Are Highly Enriched in the 65 O-GlcNAcylated Proteins Regulated by TG Administration

Compared to the saline group, out of the 506 peptides there were 85 unique peptides (corresponding to 86 O-GlcNAcylation sites) that have O-GlcNAcylation levels of >1.5 fold increase after TG (*p*-value <0.02, Supplementary Table S2). Interestingly, five peptides (corresponding to four proteins) exhibited lower O-GlcNAcylation levels (*p* < 0.02) including peptides from EMAL1, PCLO, HCFC1 and WNK1, and one PCLO and one WNK1 peptide have <50% O-GlcNAcylation after TG compared to saline (Supplementary Table S1). These results support the hypothesis that OGA activity is an important regulator of O-GlcNAcylation in the brain.

We next searched on databases to see if the 86 sites that exhibited 1.5-fold increase in O-GlcNAcylation levels have been reported or whether they are near phosphorylation sites. We found that 53 sites are known O-GlcNAcylation sites as reported on curated O-GlcNAcAtlas database (https://oglcnac.org/) (Ma et al., 2021b), 28 are reciprocal phosphorylation sites, 75 have proximal (±10 amino acids) phosphorylation sites, and 14 are not near known phosphorylation sites as reported on PhosphoSitePlus^®^ database (https://www.phosphosite.org/) (Hornbeck et al., 2015) (Supplementary Table S2).

As the 85 peptides correspond to 65 proteins, we performed pathway analyses of these 65 proteins that exhibited increased O-GlcNAcylation levels following TG treatment. We found that these proteins are primarily enriched in cytoskeleton organization, vesicle-mediated transport in synapse, and synaptic organization (Figure 1D), and that not all pathways that are linked to the 278 O-GlcNAcylated proteins are equally affected by TG.

**FIGURE 2:**
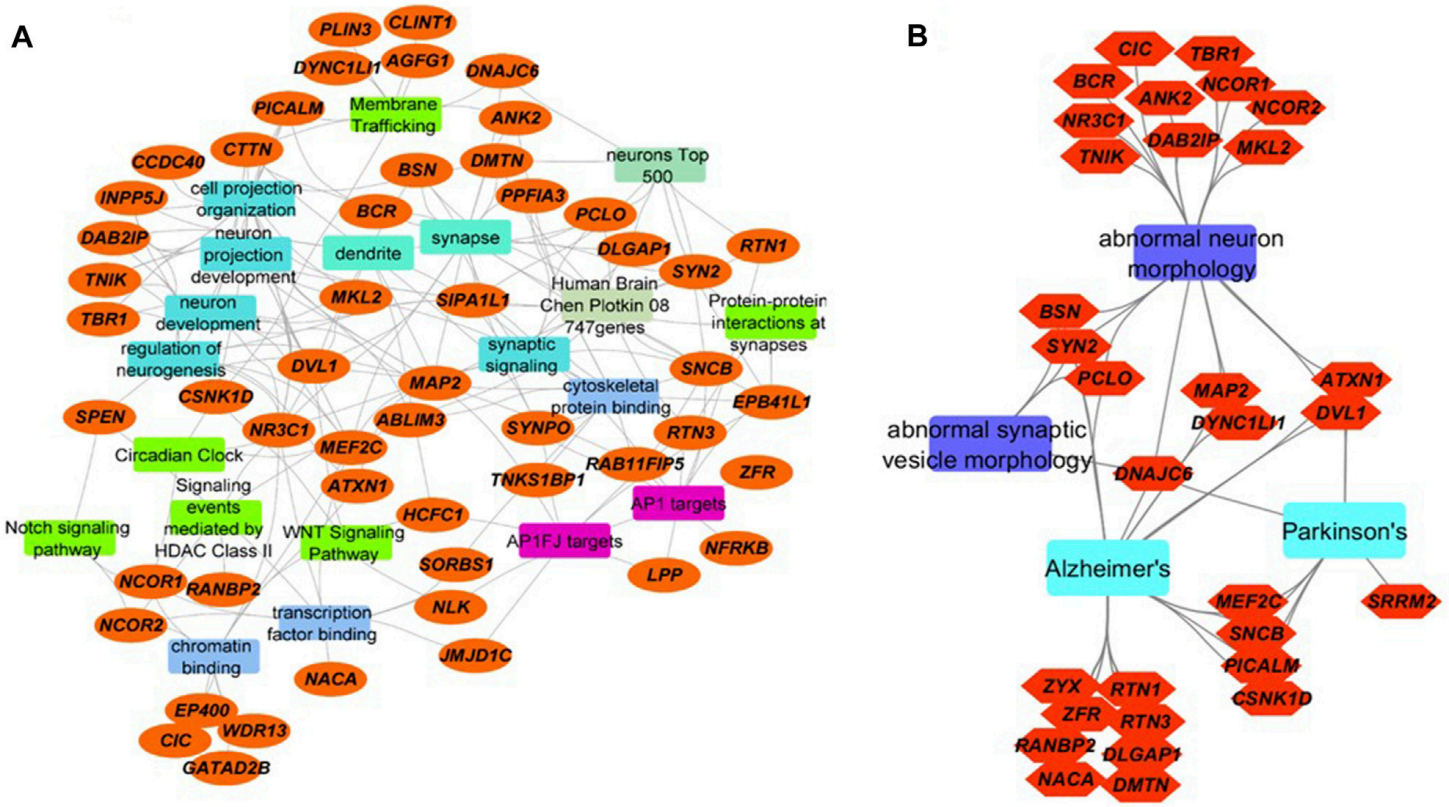
Integrated network visualizations of the involvement of the 65 proteins with significantly increased O-GlcNAcylation levels following TG administration in cellular functions, biological processes, and phenotypes **(A)** We found that 52 of the 65 proteins (orange ellipses) are involved in key pathways (green rectangles), biological processes (aqua blue rectangles), cellular functions (blue rectangles), and cellular components (jungle green rectangles). Several are targets for AP-1 family of transcription factors (purple rectangles), and many of them are in Allen Brain Atlas’s top 500-gene list and Chen-Plotkin’s 747 genes that are related to human frontotemporal lobar degeneration (other colored rectangles) **(B)** 31 of the 65 proteins (orange hexagons) are involved in abnormal neuron morphology and abnormal synaptic morphology, as well as neurodegenerative diseases including AD and PD (sky blue and violet rectangles). Eight proteins are involved both in abnormal neuron or synaptic vesicle morphology and AD. Three are involved both in abnormal neuron or synaptic vesicle morphology and Parkinson’s disease. Seven proteins are shared between AD and PD (including DNAJC6, MEF2C and PICALM).

Using protein-protein interaction data and information from multiple sources including OMIM and specialized databases related to neurodegenerative diseases (AlzGene, PDGene and SZGene) stored in the ToppGene knowledgebase (Shannon et al., 2003), we found that the 65 proteins are highly enriched for synaptic signaling, neuron projection, membrane trafficking, transcription, cytoskeletal protein binding, and circadian regulation (Figure 2A, Supplementary Table S3). Of the 65 proteins, 10 of them have cytoskeletal protein binding functions, nine proteins are associated with membrane trafficking, 18 proteins are associated with cell projection and neuron development, and 13 proteins are associated with chromatin and transcription factor binding functions.

The 10 O-GlcNAcylated proteins in the group of cytoskeletal protein binding include: MAP2, ABLIM3, SYNPO, TNKS1BP1, RAB11FIP5, RTN3, SNCB, EPB41L1, DMTN and ANK2. Most of these are also in the groups of protein-protein interactions at synapses, and synaptic signaling. The combined group of cytoskeletal protein binding, protein-protein interactions at synapses, and synaptic signaling have ∼20 proteins. Microtubule-associated protein 2 (MAP2) is proposed to be important for microtubule stabilization and may be involved in dendritic morphology (Dehmelt and Halpain, 2005). Actin binding LIM protein 3 (ABLIM3) is involved in cell-cell contacts and modulates learning and memory (Guo et al., 2018). Synaptopodin (SYNPO) may be involved in actin organization, cell motility and autophagy (Asanuma et al., 2006; Ji et al., 2019). Tankyrase-1-binding protein (TNKS1BP) regulates actin cytoskeleton rearrangement and is dysregulated both in blood and brain in AD (Ohishi et al., 2017; Pang et al., 2017). RAB11-interacting protein 5 (RABFIP5) is important for protein trafficking via binding to RAB11 GTPase (Grant and Donaldson, 2009). Reticulon 3 (RTN3) may be involved in regulation of ER autophagy, BACE1 activity and amyloid β production (Murayama et al., 2006; Grumati et al., 2017). β-synuclein (SNCB) is important for synaptic function and a negative regulator of α-synuclein aggregation (Clayton and George, 1999; Hashimoto et al., 2001). Band 4.1-like protein 1 (EPB41L1) may be involved in cortical actin cytoskeleton organization and its missense mutations were found in nonsyndromic intellectual disability patients (Hamdan et al., 2011). Dematin actin binding protein (DMTN) also plays a role in actin dynamics (Khan et al., 2008). Ankyrin 2 (ANK2) interacts with PINK2/Parkin-target proteins and ANK2 variants are associated with the risk of PD (Auburger et al., 2019).

O-GlcNAcylated proteins involved in membrane trafficking include: 1) DNAJC6, which is important for clathrin uncoating (Edvardson et al., 2012); 2) phosphatidylinositol binding clathrin assembly protein (PICALM), which can interact with LC3 and regulate amyloid precursor protein cleaved C-terminal fragment (APP-CTF) degradation (Tian et al., 2013); 3) cytoplasmic dynein one light intermediate chain 1 (DYNC1LI1) whose knockdown led to dendritic atrophy (Liu et al., 2016); 4) Perilipin 3 (PLIN3, tail-interacting protein 47 kDa, TIP47, Mannose-6-Phosphate Receptor-Binding Protein 1, M6PRBP1), which is required for endosome-to-Golgi transport and delivery of lysosomal hydrolases from the Golgi to endosomes (Diaz and Pfeffer, 1998; Carroll et al., 2001); 5) clathrin interactor 1 (CLINT1), which may be involved in clathrin coated vesicles and *trans*-Golgi network-endosome trafficking (Pimm et al., 2005); 6) ArfGAP with FG repeats 1 (AGFG1), which may be involved in both nucleocytoplasmic transport and clathrin-mediated endocytosis, 7) ankyrin2 ANK2, 8) casein kinase I delta (CSNK1D), and 9) cortactin (CTTN), which are all thought to be involved in membrane trafficking.

### Proteins Relevant to Neurodegenerative Diseases Are Dynamically Regulated by O-GlcNAcylation

Many of the 65 proteins with O-GlcNAcylation levels changed after TG administration are involved in abnormal neuron morphology and synaptic vesicle morphology, AD and PD pathogenesis (Figure 2B, Supplementary Table S3). Relevant to PD, α-synuclein in this study is O-GlcNAcylated at T72, T53 and T81 (Table 1), sites previously identified (Alfaro et al., 2012; Levine et al., 2019). As discussed above, nine proteins involved in membrane trafficking exhibit dynamic O-GlcNAcylation with O-GlcNAcylation levels sensitive to systemic TG administration. The most highly regulated O-GlcNAcylated protein by TG is DNAJC6 (with 12-fold difference between TG and saline injected mouse cortex). Mutations of DNAJC6 have been found to be responsible for juvenile and early onset PD (Edvardson et al., 2012; Köroğlu et al., 2013; Elsayed et al., 2016; Olgiati et al., 2016). Relevant to AD, one of the 65 peptides is phosphatidylinositol-binding clathrin assembly protein (PICALM) (showing a 2-fold difference between TG and saline injected mouse cortex), which is associated with late-onset AD in genome wide association studies (Harold et al., 2009). Both DNAJC6 and PICALM are involved in protein trafficking. Furthermore, MAP2 is associated with both AD and PD and is increased by 2-fold by TG at S472, but not S361 nor S788. The O-GlcNAcylation sites for DNAJC6 and PICALM that have changed O-GlcNAcylation levels by TG were previously known, while the MAP2 O-GlcNAcylation site S472 which is changed by TG was a previously un-identified site. While DNAJC6 and PICALM O-GlcNAcylation have not been investigated thoroughly, O-GlcNAcylation of MAP2 related protein tau has been extensively studied with various TG administration strategies (i.v., i.c.v., i.p., and drinking water), on multiple S/T residues, especially in transgenic mice overexpressing human tau or tau mutations (Mueller et al., 2021).

**TABLE 1:** O-GlcNAcylation sites of DNAJC6 (AUXI), PICALM, alpha-synuclein (SYUA), and MAP2 identified in the present study in comparison with the two cortical O-GlcNAcome studies.

Protein	Gene name	Study	Subject	Accession number	O-GlcNAcylation sites/regions
AUXI	DNAJC6	Present study	Mouse	Q80TZ3	S591
—	—	Alfaro et al	Mouse	Q80TZ3	V600-R640
—	—	Wang et al	Human	Not detected	Not detected
PICAL	PICALM	Present study	Mouse	Q7M6Y3	S453
—	—	Alfaro et al	Mouse	Q7M6Y3	K349-K388, S443-R461
—	—	Wang et al	Human	Q13492	S565, T291
SYUA	SCNA	Present study	Mouse	O55042	T53, T72, T81
—	—	Alfaro et al	Mouse	O55042	T53, T64, T72
—	—	Wang et al	Human	P37840	T54, T64, T72, T81
MTAP2	MAP2	Present study	Mouse	P20357	S361, S472, S788
—	—	Alfaro et al	Mouse	P20357	T466-K483, V776-K795
—	—	Wang et al	Human	P11137	S199, T241, T335, S502, T522, T851, S1082

Related to synaptic pathway function, acute TG injection in rats impairs learning and memory (Taylor et al., 2014), in rat hippocampal slices induces long-term depression (Taylor et al., 2014), in rodent hippocampal slices attenuated epileptiform activity at CA3-CA1 synapses as well as spontaneous CA3 pyramidal cell activity (Stewart et al., 2017), and in rodent hippocampal slices decreased GABA_A_R currents and intrinsic excitability (Stewart et al., 2020). O-GlcNAcylation has been shown to regulate cellular clock oscillation, the level and stability of clock regulators (Li et al., 2013), as perturbation of the sleep/wake cycle occurs in neurodegenerative disease patients, O-GlcNAcylation of circadian regulators may be important for disease pathogenesis and/or therapeutic considerations (Austad et al., 2021).

### Unique and Common Proteins Identified Compared to Two Extensive Cortical O-GlcNAcome Studies

**FIGURE 3:**
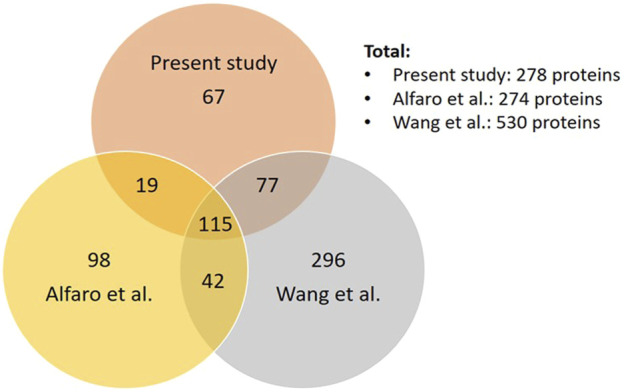
Comparison of our dataset to the two most extensive datasets of cortical O-GlcNAcome. Venn diagram showing the number of proteins that are unique or common among our current datasets, Alfaro et al. (Alfaro et al., 2012) and Wang et al. (Wang et al., 2017).

Comparing our dataset with the two most extensive studies on O-GlcNAcome in the mouse (274 proteins) and human (530 proteins) cortex (Alfaro et al., 2012; Wang et al., 2017), we found that there are a total of 714 proteins identified combining our current and the two prior studies, with 115 in common and 67 unique proteins in our study (Figure 3, Supplementary Figure S1). The 115 proteins shared among the three datasets include cell junction proteins BSN and PCLO, which is expected since they are abundant and possess numerous O-GlcNAcylation sites **(** Supplementary Figure S1A **)**. There are also proteins involved in cellular component organization and morphogenesis processes, such as cytoskeleton proteins ABLM2, ANK2, and MAP1B; synaptic proteins SYN1, SYN2, SYNPO, SYUA, and SYUB. Among synaptic signaling proteins, ULK2 and STAT3 are regulators of autophagy. The 67 proteins unique to the present study are also involved in cellular component organization and morphogenesis processes. These include synapse organization proteins such as DAB2IP, NPTN, and CTTNBP2 and localized in dendrite and microtubule components **(** Supplementary Figure S1B **)**. They also include HDAC6 a histone deacetylase; signaling proteins INP4A and INPP; and LRP5 which is involved in endocytosis, mTOR signaling and AD. DNAJC6/AUXI has been shown to be O-GlcNAcylated in both mouse studies but not in human postmortem mid frontal gyrus O-GlcNAcome. Potential autophagy regulators including MAP1A, MEF2C, NUP62, TFE3 and TPPP are present in both the human O-GlcNAcome and our current study but not in the prior mouse cerebrocortical tissue study. Although the Alfaro study also used mouse cortex, they used 1 year old female mice (1 each WT and 3xTg AD model). In contrast, our study used male C57BL/6J strain 2–3 each WT with Saline or Thiamet G injection at 2 months of age. Additionally, the TMT labeling might change the ionization efficiency of some peptides. These differences likely contribute to the differences in the O-GlcNAc proteome observed between our studies.

We also compared the 65 proteins with O-GlcNAcylation levels significantly increased by TG with the 81 proteins with O-GlcNAcylation levels significantly changed in human AD brains versus control brains (Wang et al., 2017). We found 15 proteins in common, which are ANK2, EPN4, EMSY, EP400, P66B, HCFC1, JHD2C, GCR, PRC2C, QRIC1, RBP2, MINT, SYNPO, ZFR, and ZYX (Supplementary Table S4). All of these proteins showed higher O-GlcNAcylation levels in AD brains compared to matched controls except for SYNPO, which showed lower levels in AD brains, and has been suggested to be involved in actin organization, cell motility and autophagy (Grant and Donaldson, 2009; Grumati et al., 2017). In addition, six of these 15 proteins are enriched in chromatin organization process, which include EP400, HCFC1, JMJD1C, EMSY, GATAD2B, and NR3C1. HCFC1 loss of function resulted in brain development deficits (Jolly et al., 2015). EP400 is required for oligodendrocyte survival (Elsesser et al., 2019). NR3C1 is a nuclear receptor of the steroid/thyroid/retinoic acid superfamily and whose methylation has been found to be involved in stress response (Oberlander et al., 2008). JMJD1C is involved in mitochondrial dysfunction in response to MPP+ in SH-SY5Y cells (Wang et al., 2016). GATAD2B is associated with neurodevelopmental disorders and intellectual disabilities (Willemsen et al., 2013; Kaur et al., 2019; Trubnykova et al., 2019; Ueda et al., 2019; Shieh et al., 2020a; Shieh et al., 2020b; Vera et al., 2020). There are 50 proteins that are changed with their O-GlcNAcylation by TG in our study, but the change of their O-GlcNAcylation in AD compared to control has not reached significance. This include MAP2, the abundance of which is increased in AD (with log2∼05 and q∼0.01) but not O-GlcNAcylation, and PICALM (Wang et al., 2017). Whether the O-GlcNAcylation of these proteins is linked to AD phenotypes and whether modulating their O-GlcNAcylation after AD pathogenesis can impact disease progression is an important question for future studies.

## Summary

In this study we first assessed the impact on total levels of protein O-GlcNAcylation in response to TG using the lower resolution western blotting techniques and found a robust 3-fold increase in protein O-GlcNAcylation (Figure 1). Using the isobaric tandem mass tag labelling combined with chemoenzymatic photocleavage (CEPC) method we examined the O-GlcNAc proteome with and without the i. p administration of the inhibitor of OGA TG (Wang et al., 2017). This approach allowed us to characterize the O-GlcNAc proteome and those sensitive to acute inhibition of OGA. We identified 506 O-GlcNAcylated peptides, and of these, 85 peptides corresponding to 65 proteins were at least 50% more O-GlcNAcylated from the TG treated mouse cortex compared to saline control. Of those sensitive to OGA inhibition the O-GlcNAcylated proteins were highly enriched for synaptic signaling, neuron projection, membrane trafficking, transcription, and cytoskeletal protein binding. The most regulated protein DNAJC6 is associated with the risk for PD, raising the question whether its O-GlcNAcylation may contribute to pathogenesis. MAP2, which is associated with both AD and PD, and PICALM which is associated with AD are also regulated by TG by 2-fold.

One important feature of this study is that the observed increase in the levels of O-GlcNAcylation of 85 peptides corresponding to 65 protein (5 peptides corresponding to four proteins exhibited decreased levels of O-GlcNAcylation) occurred within only 3 h of TG administration. These peptides/proteins are likely the most sensitive to changes of OGA/OGT activities and/or substrates in response to intracellular and extracellular stimuli. It is also of interest that pharmacological inhibition of OGA affects a sub-O-GlcNAc proteome suggesting the potential for selectivity in its mechanisms of action. Despite that these highly dynamic changes are likely to be the “first responders.” they have been often ignored in studies of pathologies associated with altered levels of O-GlcNAcylation. The numbers of O-GlcNAc modification sites on a given protein range from 0 to over 50 on different proteins. The implications for the impact of the O-GlcNAcylation pathway on the network of pathway in the brain is still poorly understood. Determining how different O-GlcNAcylated proteins in different regions of the brain impact neuronal function and survival will be important for understanding cognition and neurodegeneration. Future studies will define the role of O-GlcNAcylation in neuronal function as well as in aging and neurodegenerative disease pathogenesis.

## Data Availability

The raw datasets presented in this study can be found in online repositories. The names of the repository/repositories and accession number(s) can be found below: Massive.ucsd.edu with accession: MSV000088053. The data will also be available through ProteomeXchange with accession: PXD028204.
