# Supplementary material for: Defining the Dynamic Regulation of O-GlcNAc Proteome in the Mouse Cortex---the O-GlcNAcylation of Synaptic and Trafficking Proteins Related to Neurodegenerative Diseases
Source: Front Aging. 2021 Sep 29;2:757801. doi: 10.3389/fragi.2021.757801 (PMC9261315; doi:10.3389/fragi.2021.757801)
Supplement: Supplementary file 3 [file Presentation1.pptx]

## Slide 1
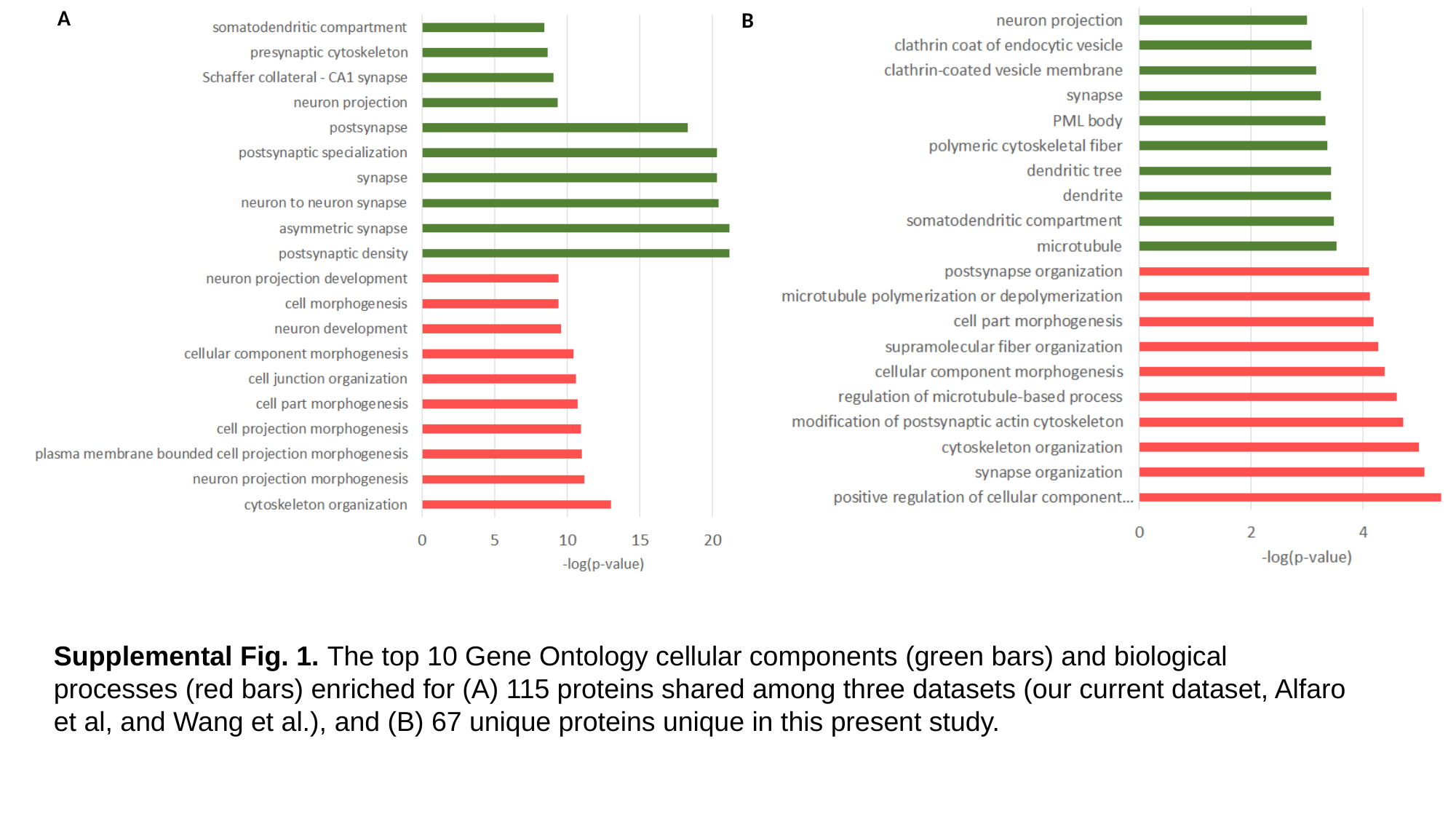

A
B
Supplemental Fig. 1. The top 10 Gene Ontology cellular components (green bars) and biological processes (red bars) enriched for (A) 115 proteins shared among three datasets (our current dataset, Alfaro et al, and Wang et al.), and (B) 67 unique proteins unique in this present study.

## Slide 2
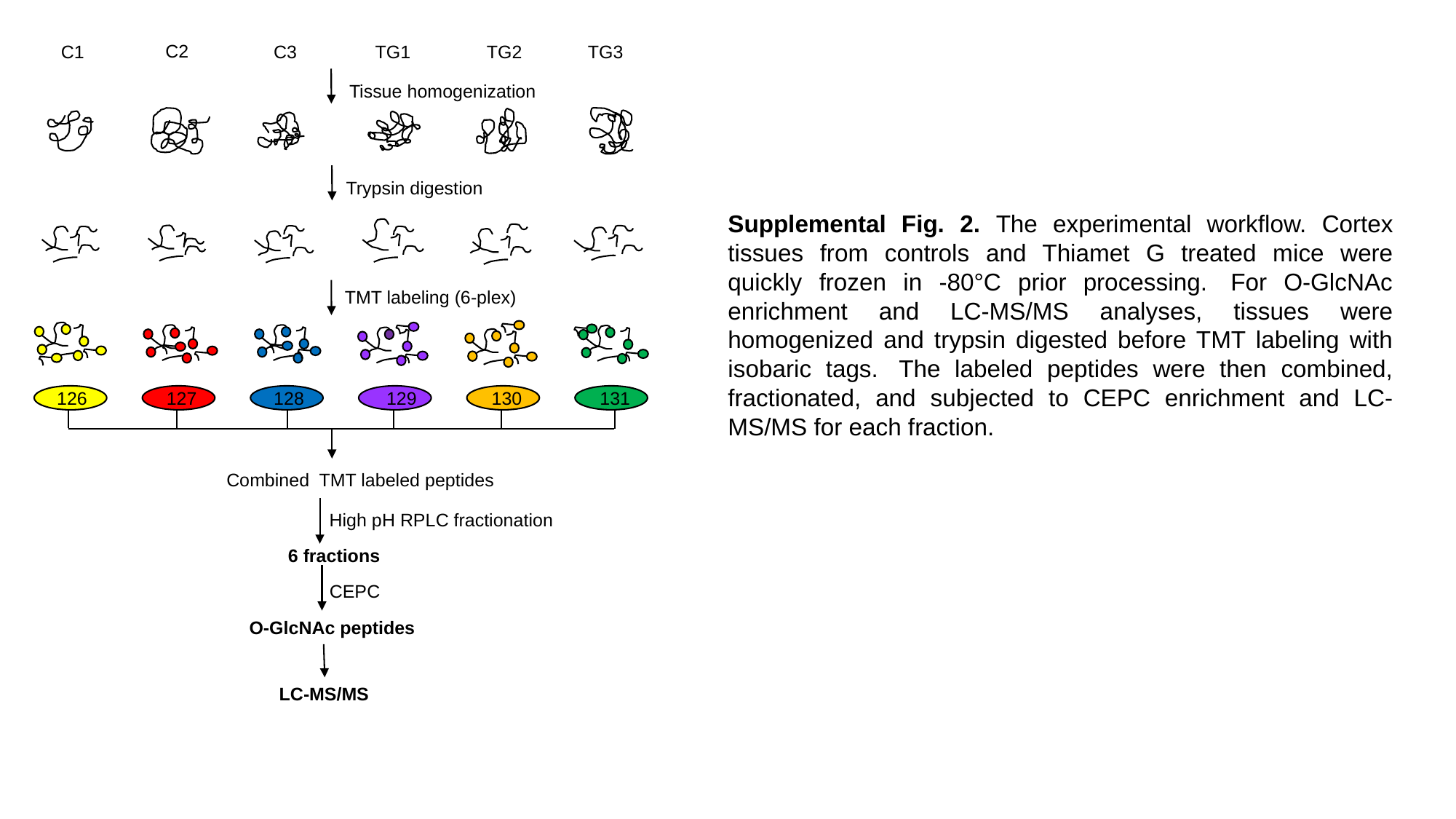

C2
C1
C3
TG1
TG2
TG3
Tissue homogenization
Trypsin digestion
TMT labeling (6-plex)
126
127
128
129
130
131
Combined TMT labeled peptides
High pH RPLC fractionation
6 fractions
CEPC
O-GlcNAc peptides
LC-MS/MS
Supplemental Fig. 2. The experimental workflow. Cortex tissues from controls and Thiamet G treated mice were quickly frozen in -80°C prior processing.  For O-GlcNAc enrichment and LC-MS/MS analyses, tissues were homogenized and trypsin digested before TMT labeling with isobaric tags.  The labeled peptides were then combined, fractionated, and subjected to CEPC enrichment and LC-MS/MS for each fraction.

## Slide 3
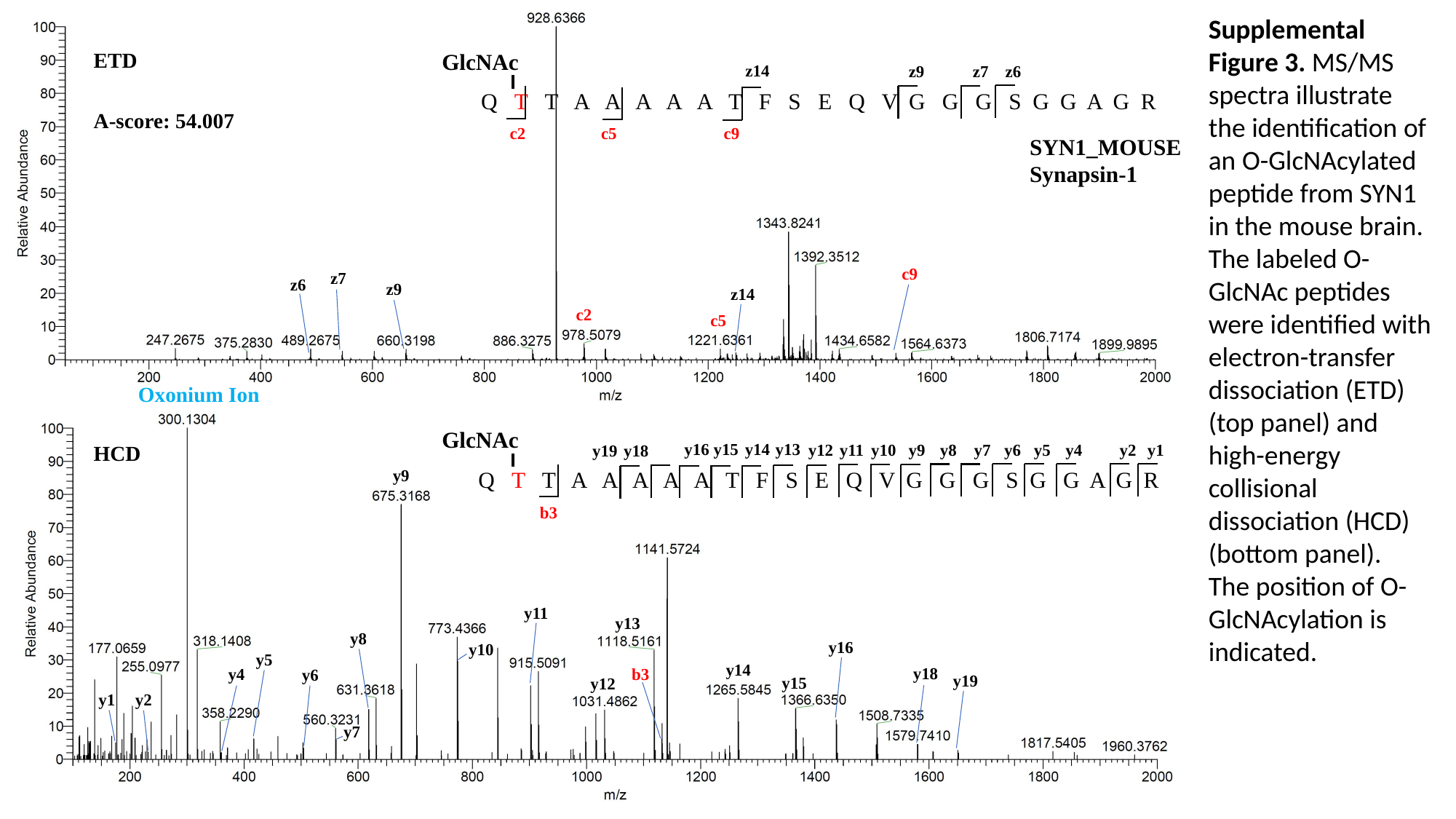

Supplemental Figure 3. MS/MS spectra illustrate the identification of an O-GlcNAcylated peptide from SYN1 in the mouse brain.  The labeled O-GlcNAc peptides were identified with electron-transfer dissociation (ETD) (top panel) and high-energy collisional dissociation (HCD) (bottom panel).  The position of O-GlcNAcylation is indicated.
ETD
GlcNAc
y16 y15 z14 y13
y12 y11 y10 z9 y8 z7 z6 y5 y4 y3 y2 y1
Q T T A A A A A T F S E Q V G G G S G G A G R
A-score: 54.007
b10
c2 c3 b4 c5 b6 b7 b8 c9
SYN1_MOUSE
Synapsin-1
c9
z7
z6
z9
z14
c2
c5
Oxonium Ion
GlcNAc
y16 y15 y14 y13
y12 y11 y10 y9 y8 y7 y6 y5 y4 y3 y2 y1
HCD
y19 y18
y9
Q T T A A A A A T F S E Q V G G G S G G A G R
b10
b2 b3 b4 b5 b6 b7 b8
y11
y13
y8
y16
y10
y5
y14
y18
y4
b3
y6
y19
y15
y12
y1
y2
y7
