# Supplementary material for: Defining the Dynamic Regulation of O-GlcNAc Proteome in the Mouse Cortex---the O-GlcNAcylation of Synaptic and Trafficking Proteins Related to Neurodegenerative Diseases
Source: Front Aging. 2021 Sep 29;2:757801. doi: 10.3389/fragi.2021.757801 (PMC9261315; doi:10.3389/fragi.2021.757801)
Supplement: Supplementary file 6 [file DataSheet1.docx]

**Supplemental Information**

**Method:**

**Mice:** All animal experiments were approved by University of Alabama at Birmingham IACUC. C57BL/6 male mice at 2 months of age were i.p. injected with saline or TG at 10 mg/kg. The mouse cortex was dissected 3 hours later, and flash frozen in liquid nitrogen and stored at -80°C for western blot (n=3 each control versus TG) and proteomics assays (from 3 control mice and 2 TG treated mice).

**Western blot analyses:** Mouse cortex samples were lysed in lysis buffer with 50 mM Tris pH 7.4, 175 mM NaCl, 5 mM EDTA pH 8.0. Western blots were performed using O-GlcNAc (UAB hybridoma core, CTD110.6, 1:10,000) and GAPDH (Millipore, MAB374, 1:5000) antibodies. Specificity of the antibody for O-GlcNAcylated proteins in this protocol was verified by competition of the label with N-acetyl Glucosamine. GraphPad Prism 8.2.1. (GraphPad software, Inc, La Jolla, CA) was used for statistical analyses. ImageJ was used for density quantification. A value of *p* < 0.05 was considered significant.

Tissue homogenization and protein digestion, TMT labelling, and fractionation. Cortex tissues (~30 mg) was homogenized in 600 μL of lysis buffer (8 M urea, 60 mM NH_4_HCO_3_, 1x HALT phosphatase inhibitor cocktail (Pierce), 20 μM PUGNAc and 10 mM dithiothreitol (DTT). After homogenization, proteins were reduced and alkylated by incubated at 37 ˚C for 1 followed by another 30 min with 25 mM Iodoacetamide (IAA). The sample was then diluted 4-fold with 50 mM NH_4_HCO_3_, 2 mM CaCl_2_, and digested with trypsin (1:50 enzyme-to-substrate ratio), first for 3 h at 37˚C, then overnight at room temperature after being further diluted one fold and same amount of trypsin added. After digestion, the solution was acidified with 10% trifluoroacetic acid ‎(TFA) and centrifuged at 14,000 g for 20 min before desalted by C18 solid phase extraction (SPE) and concentrated using Speed-Vac. Peptide concentration was determined with BCA assay (Pierce) (Supplemental Figure 2).

Peptides from control and mice treated TG (n=3 for each group) were used for isobaric TMT labelling. ~500 μg of peptides from each sample were dried down and dissolved in 180 μL of 250 mM sodium borate, pH 8.6, before 5 mg of TMT-6 in 430 µL acetonitrile (ACN) was added. The detailed labeling and mixing information in the TMT-6 experiments was similar as previously described (Wang et al., 2017). The reaction was allowed to carry on at RT for 90 min before terminated with 5% hydroxylamine. Samples with different TMT labeling in the same TMT-6 experiment were combined and desalted on C18 SPE column. The combined peptides were fractionated on a 250 x 4.6 mm C18 column by Agilent 1200 HPLC System (Agilent) using the basic pH RPLC method and concatenated into 6 final fractions (Wang et al., 2011) for enrichment of O-GlcNAc (**Supplemental Figure 2**).

**Enrichment of O-GlcNAc.** Enrichment of O-GlcNAc followed the CEPC method as previously described (Alfaro et al., 2012; Wang et al., 2010) with minor modifications. The fractionated peptides (~300 μg) were dried in vacuum and re-suspended in 1.1 mL of 20 mM HEPES, pH 7.9, and 80 μL of 100 mM MnCl_2_, 75 μL of 0.5 mM UDP-GalNAz, 62 μL of GalT1 and 2.5 μL of PNGase F (500 U/μL, New England Biolabs) were added. The mixture were incubated at 4 ˚C for ~20 h, and then at RT for 3 h. After this glycotransfer step, the peptides were reacted with 1 mM biotin-PEG-PC-alkyne (Ambergen) in 20 mM HEPES, pH 7.9 using 0.8 mM CuSO_4_, 4 mM tris(3-hydroxypropyltriazolylmethyl)amine (THPTA), 20 mM sodium ascorbate, 20 mM aminoguanidine as the catalyst for copper catalysed alkyne-azide cycloaddition (CuAAC, or click chemistry) (Hong et al., 2009). Extra reagents were removed by C18 SPE. The peptides were neutralized with PBS and incubated with NeutrAvidin agarose resin (Pierce) for 2 h at RT, and washed thoroughly with 2 M Urea in PBS (6x), 4 M Urea in 50 mM NH_4_HCO_3_ (6x), 2 M NaCl (6x), water (1x), and 70% MeOH (5x). In the end, the O-GlcNAc peptides were released from the resin by 365 nm UV radiation for 25 min (**Supplemental Figure 2**).

**LC-MS/MS and data analysis.** The enriched O-GlcNAc peptide samples were injected and analysed using a Waters nanoAquity UPLC system coupled online to a LTQ Orbitrap Velos MS (Thermo Scientific) as previously described (Wang et al., 2017). Samples were separated on a home-made column (75 μm inner diameter, 70 cm long, packed with 3μm C18 particles (Phenomenex) with 1) a 100-min gradient. Full MS spectra (400-2,000 m/z) were acquired with a resolution of 60,000. Top 10 most intensive precursor ions were selected for MS/MS using alternating ETD and HCD. One hundred ms activation time and supplemental activation were used in ETD, and the product ions were detected in an ion trap. Thirty-two percent normalized collision energy was used in HCD fragmentation, and the product ions were detected in the Orbitrap with a resolution of 7,500.

Data were searched against a UniProt protein database (version 2015_04) in the decoy mode using MS-GF+ (v9881)(Kim and Pevzner, 2014). The searching parameters were: precursor ion mass tolerance (±10 ppm), partial tryptic specificity, dynamic oxidation of Met (15.9949 Da), static alkylation on Cys (57.0215 Da), static TMT6 labelling on N-terminal and Lys (229.1629 Da), and dynamic O-GlcNAcylation on Ser and Thr (502.2023 Da, GalNAz labelled and photocleaved). The search results from ETD spectra were first filtered by Q value <0.01 to obtain a false discovery rate (FDR) of <1% at the peptide level. The identification of an O-GlcNAc peptide was then confirmed/filtered by the presence of the diagnostic 300.13 m/z ion in subsequent corresponding HCD spectra(Alfaro et al., 2012). The Ascore (Beausoleil et al., 2006) was used to estimate the confidence and adjust the site assignment for O-GlcNAcylation. An Ascore of >13 was required for a confident site assignment (*p* <0.05). The intensities of all six TMT reporter ions were extracted using MASIC software (Monroe et al., 2008) (**Supplemental Figure 2**). A representative MS/MS spectra is shown in **Supplemental Figure 3**.

The raw datasets presented in this study can be found in online repositories. The names of the repository/repositories and accession number(s) can be found below: [Massive.ucsd.edu](https://urldefense.com/v3/__http:/massive.ucsd.edu/__;!!NoSwA-eRAg!RQqgTsEvpUi1IVmyWJcCIxjlo4gZP3ffOyFVmf7RZj2glmgMALKHs5HTgciNKzGLCB8z$) with accession: MSV000088053. The data will also be available through ProteomeXchange with accession: PXD028204.

**Functional enrichment analysis, comparative network visualization, and post-translational modification site search:** We examined protein lists for relative enrichment of corresponding genes associated with Gene Ontology, pathways, mouse phenotypes, and human diseases. We used the ToppFun program of the ToppGene Suite for this purpose (Chen et al., 2009; Chen et al., 2007). Briefly, gene ontology annotations from NCBI, pathway annotations from several resources, and disease-gene data from multiple sources including OMIM and specialized databases related to neurodegenerative diseases (AlzGene, PDGene and SZGene) in the ToppGene knowledgebase were used for functional enrichment analysis (for details refer to (Chen et al., 2009; Chen et al., 2007)). We used protein-protein interaction data and Cytoscape, a *JAVA*-based bioinformatics software package for visualizing and analyzing molecular and genetic interaction networks (Ideker et al., 2002; Shannon et al., 2003; Spirin and Mirny, 2003). The enrichment results from the ToppGene Suite were converted into Cytoscape-compatible files and loaded into Cytoscape along with attribute files for network visualization. We analyzed whether the O-GlcNAcylation sites are reported glycosylation sites, known phosphorylation site, or near known phosphorylation site using PhosphoSitePlus database (<https://www.phosphosite.org/>) and O-GlcNAcAtlas (https://oglcnac.org/).

Alfaro, J.F., Gong, C.-X., Monroe, M.E., Aldrich, J.T., Clauss, T.R., Purvine, S.O., et al. (2012). Tandem mass spectrometry identifies many mouse brain O-GlcNAcylated proteins including EGF domain-specific O-GlcNAc transferase targets. Proceedings of the National Academy of Sciences *109*, 7280-7285.

Beausoleil, S.A., Villén, J., Gerber, S.A., Rush, J., and Gygi, S.P. (2006). A probability-based approach for high-throughput protein phosphorylation analysis and site localization. Nature biotechnology *24*, 1285-1292.

Chen, J., Bardes, E.E., Aronow, B.J., and Jegga, A.G. (2009). ToppGene Suite for gene list enrichment analysis and candidate gene prioritization. Nucleic Acids Res *37*, W305-W311.

Chen, J., Xu, H., Aronow, B.J., and Jegga, A.G. (2007). Improved human disease candidate gene prioritization using mouse phenotype. BMC. Bioinformatics *8*, 392.

Hong, V., Presolski, S.I., Ma, C., and Finn, M. (2009). Analysis and Optimization of Copper‐Catalyzed Azide–Alkyne Cycloaddition for Bioconjugation. Angewandte Chemie International Edition *48*, 9879-9883.

Ideker, T., Ozier, O., Schwikowski, B., and Siegel, A.F. (2002). Discovering regulatory and signalling circuits in molecular interaction networks. Bioinformatics *18 Suppl 1*, S233-S240.

Kim, S., and Pevzner, P.A. (2014). MS-GF+ makes progress towards a universal database search tool for proteomics. Nat Commun *5*, 5277.

Monroe, M.E., Shaw, J.L., Daly, D.S., Adkins, J.N., and Smith, R.D. (2008). MASIC: a software program for fast quantitation and flexible visualization of chromatographic profiles from detected LC-MS(/MS) features. Computational biology and chemistry *32*, 215-217.

Shannon, P., Markiel, A., Ozier, O., Baliga, N.S., Wang, J.T., Ramage, D., et al. (2003). Cytoscape: a software environment for integrated models of biomolecular interaction networks. Genome Res *13*, 2498-2504.

Spirin, V., and Mirny, L.A. (2003). Protein complexes and functional modules in molecular networks. Proc. Natl. Acad. Sci. U. S. A *100*, 12123-12128.

Wang, S., Yang, F., Petyuk, V.A., Shukla, A.K., Monroe, M.E., Gritsenko, M.A., et al. (2017). Quantitative proteomics identifies altered O-GlcNAcylation of structural, synaptic and memory-associated proteins in Alzheimer's disease. J Pathol *243*, 78-88.

Wang, Y., Yang, F., Gritsenko, M.A., Wang, Y., Clauss, T., Liu, T., et al. (2011). Reversed‐phase chromatography with multiple fraction concatenation strategy for proteome profiling of human MCF10A cells. Proteomics *11*, 2019-2026.

Wang, Z., Udeshi, N.D., O'Malley, M., Shabanowitz, J., Hunt, D.F., and Hart, G.W. (2010). Enrichment and site mapping of O-linked N-acetylglucosamine by a combination of chemical/enzymatic tagging, photochemical cleavage, and electron transfer dissociation mass spectrometry. Molecular & Cellular Proteomics *9*, 153-160.
